# Effects of the COVID-19 pandemic on life scientists

**DOI:** 10.1186/s13059-020-02031-1

**Published:** 2020-05-11

**Authors:** Jan O. Korbel, Oliver Stegle

**Affiliations:** 1 European Molecular Biology Laboratory, Genome Biology Unit, Heidelberg, Germany; 2 Divison of Computational Genomics and Systems Genetics, German Cancer Research Center (DKFZ), Heidelberg, Germany

We will not know the long-term impact of the SARS-CoV-2 viral outbreak for some time yet, but many of us have already begun to feel the effects—not only on our daily lives but also on our work as life scientists. With partial or complete institutional shutdowns in countries worldwide, the global COVID-19 health crisis has rapidly impacted the life science landscape, including our patterns of work. Some life scientists may today feel essentially “stuck,” unable to carry out experiments because of COVID-19-related working restrictions or because they need to look after children in connection with the closure of schools and kindergartens. This can be a frightening feeling, especially for young life scientists, who usually have short-term contracts and may worry about their future careers.

Other scientists may have begun using the times of shutdowns and curfews to develop scientific projects further while working from home. In fact, Isaac Newton developed the essence of his groundbreaking scientific work during a pandemic when he was forced to work from home due to a plague outbreak in 1665, when the University of Cambridge sent its students home to continue their studies. For Newton, this meant Woolsthorpe Manor, the family estate about 60 miles northwest of Cambridge, where he was isolated for over a year. On his return to Cambridge in 1667, he had developed his seminal theories on classical mechanics as a student working from home [1]. Only 2 years later, he became a professor at the University of Cambridge.

Of course, a lot has changed since the seventeenth century. Science today is international, globally connected, and increasingly collaborative. There are means to work from home on the computer while connecting with colleagues locally and globally using a wide range of video conferencing (VC) systems, teleconferencing platforms, or collaboration tools such as Slack [2]. For computational biologists and data scientists, collaborations can be facilitated through electronic means of communicating analysis results or co-development of computational code. The fact that nearly all communication these days is electronic also spurs new collaborations and online activities, such as virtual journal clubs held internationally, virtual scientific seminars, and ad hoc workshops and training activities on topics of common interest. Scientific conferences are increasingly held as “virtual meetings,” such as the international EMBO | EMBL Symposium “The four dimensional genome – Virtual” (normally taking place in Heidelberg, Germany) and the 2020 edition of “The Biology of Genomes” (normally held at the Cold Spring Harbor Laboratory, NY). They were run online in March and May 2020, respectively, with real-time streaming of talks and moderated live discussions.

The ability to rapidly connect with scientists in spite of institutional shutdowns has, on top of this, facilitated the engagement of researchers in collaborative activities targeted against COVID-19. This includes studies pertaining to the biology and evolution of SARS-CoV-2, pathogenesis and epidemiology of the disease, host response and host genetics, and potential therapies [3–5]. The European COVID-19 Data Portal [6], announced by the European Commission President Ursula von der Leyen on 20 April 2020, for example, is setting out to help scientists coordinate the sharing of research data related to the fight against COVID-19 using the European Open Science Cloud. Additional activities include new global efforts that aim to sequence SARS-CoV-2 viral genomes along with patient-matched host genomes or to utilize existing cohorts such as the UK Biobank [7], in order to dissect the role of host genetics in the COVID-19 disease course. On top of this, new international platforms such as Crowdfight COVID-19 [8] or data against COVID-19 [9] aim to empower scientists to work together in fighting SARS-CoV-2, by connecting expertise from different fields with data resources. Another example is a regular workshop series hosted by the European Laboratory of Intelligent Systems (ELLIS), which seeks to connect the expertise of leading researchers in machine learning and artificial intelligence for the fight against COVID-19.

In this editorial, we report on the impact of COVID-19 on the daily lives of life scientists, irrespective of whether they engage in COVID-19-related research activities or not. We focus on how the current health crisis has affected patterns of work in the life sciences and highlight who in the life science community may be particularly vulnerable in the current situation. We based a large part of this editorial on a survey that we circulated among colleagues in Germany, Spain, the UK, Italy, France, Canada, Turkey, and the USA between 15 and 23 April 2020. In total, we received 881 responses, 72% of which were from trainees, 11% from support staff, and 17% from professors. Sixty-two percent of the respondents characterized themselves as experimentalists, 34% as computational biologists, and 4% as administrative support personnel.

## Impact on life scientists: research progress and working conditions

Seventy-seven percent of the respondents stated that their institute has been fully shut down, with only essential services staff present on site. Nineteen percent reported a partial shutdown (where the institute is < 50% operational), and the remaining reported a basically “fully operational” institution.

Our survey confirmed that, overall, there has been a significant impact of institute closures on life scientists: 57% of life scientists reported that they had lost some of their work. This is likely to result in financial consequences, as repetition of work will consume additional funding. Twenty-five percent of respondents reported at least 1 month and up to 6 months of work had been lost due to laboratory shutdown—with large differences seen between wet lab (73%) and dry lab (31%) researchers. At the same time, levels of self-perceived productivity dropped, where dry lab scientists were much more likely to continue carrying out their work from home as expected (29% of dry lab scientists, but only 10% of wet lab scientists, reported “at least 80% productivity”). There was also a more pronounced increase in self-perceived levels of stress (during times of lockdown compared to before) among wet lab scientists, with higher increases seen in trainees and non-tenured professors. On the other hand, some respondents reported that their stress during the laboratory shutdown was lower than during their normal work routine, which could be explained by less frequent interruptions in their daily routine or perhaps reduced expectations from peers and lab heads to deliver results.

The personal living conditions—for example, alone versus living with a spouse or family—and whether scientists are based in an institution within their home country or whether they are expatriates also seem to affect the level of personal impact the COVID-19 outbreak has caused. We observed some differences between male and female scientists, with females reporting fewer productive hours. This is, in part, due to the higher rate of females among wet lab scientists (70% of female versus 60% of male respondents work primarily experimentally) and likely also reflects differences in childcare duties. This suggests a particular vulnerability of female scientists during an institutional shutdown. Another vulnerable group appears to be expatriates, especially trainees working in a life sciences institute located on a continent other than the one where their home country is. Thirty-four percent of these young scientists live alone, compared with only 14% of respondents working in their home country. These expatriates might feel more isolated and may also face potential problems with frequent updates to local regulations due to language difficulties.

## Educational opportunities and e-conferences

There has also been a varied impact of COVID-19 on the scientific system with respect to patterns of scientific communication, collaboration, and training. At all career stages, VC has gained importance in running group meetings or journal clubs and to meet collaborators. More than 90% of life scientists at all career stages reported in our survey that they were now more regularly making use of VC for these purposes. Nearly half of the respondents stated that their level of communication with their supervisor, mentor, or line manager had not changed (48%), whereas one fifth said it even increased (22%), which suggests that VC is fortunately heavily used and appears to be an effective means of communication and mentoring.

At the time of our survey, 30% of life scientists have attended virtual scientific conferences since the COVID-19 pandemic started, suggesting that e-conferences are becoming an important format for scientific meetings. Especially trainees reported that they have been making great use of opportunities for e-learning, including VC-based bioinformatics courses, with a higher rate of wet lab (72%) than dry lab (50%) trainees benefiting from e-learning during an institutional shutdown. This indicates that especially trainees normally based in the wet lab use the time of shutdown to expand their skillsets. This includes many young scientists now learning programming languages. For them, there is an opportunity to use the unintended break in their experimental work to develop into more interdisciplinary hybrid (wet/dry) data scientists.

## Patterns of collaboration during the COVID-19 outbreak

**Fig. 1:**
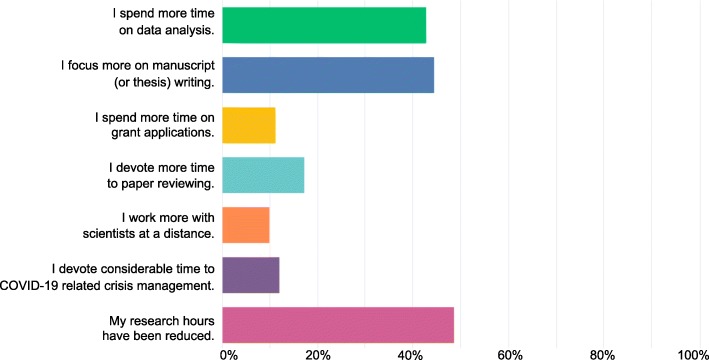
How the COVID-19 pandemic affected patterns of work of life scientists. Shown are summary statistics from self-reported data based on a survey with 881 individual responses

Although 49% of scientists reported that their research hours have been reduced during the COVID-19 outbreak, many indicated that they are using the times of shutdown to devote more time to data analysis (43%), manuscript or thesis writing (45%), or developing grant applications (11%) (see Fig. 1). Indeed, there are early signs that manuscript submissions to scientific journals have already been increasing since COVID-19-related restrictions have emerged [4]. And somewhat impressively, over a hundred respondents (102, 18% of the total) indicated that they shifted the regular scientific activities to be able to directly contribute to research with the aim to combat COVID-19. A recent inquiry circulated among members of the European Molecular Biology Organization (EMBO) provides an independent testament of the willingness of life scientists to contribute to fighting COVID-19 (https://www.data-against-covid.org). This study also showed that life scientists actively support virologists, epidemiologists, and health care workers by contributing reagents, instruments, protective equipment, and IT infrastructure (such as high-performance as well as cloud computing platforms) or by providing clinical tasks and communicating with journalists and the public (https://www.embo.org/news/articles/2020/life-science-researchers-efforts-to-fight-covid-19).

New means of collaboration appear to gain in importance for scientific research during the period of COVID-19 lockdown. Not surprisingly, 94% of life scientists completing our survey reported ample use of VC to collaborate, discuss, and develop science. Eighteen percent of computational biologists indicated that collaborative work using community software development platforms, such as provided by GitHub, gained relevance for their daily work.

Finally, although we did not explicitly ask for this in our survey, it has become clear from our own research groups and from talking to colleagues that scientists are also actively using times of social distancing to “socialize from a distance,” which includes cooking clubs, tea or coffee times, paper acceptance celebrations, and even social beer hours run via VC.

In summary, COVID-19 had substantial effects on scientists, causing stress and work interruptions, but we also see new patterns of local and international cooperation, idea exchange, and electronic learning appearing. If there is a silver lining to the current global health crisis, it would be desirable that some of these new practices are maintained and further developed once we are able to return to “business as usual” in the future. The ability to work efficiently from home, and to collaborate productively with life scientists and clinicians nationally and internationally, without extensive travel (and the associated carbon footprint) might, ultimately, even result in benefits for scientific communities and society as a whole.
